# Clinical Relevance of Plasma Prostaglandin F_2α_ Metabolite Concentrations in Patients with Idiopathic Pulmonary Fibrosis

**DOI:** 10.1371/journal.pone.0066017

**Published:** 2013-06-11

**Authors:** Kensaku Aihara, Tomohiro Handa, Toru Oga, Kizuku Watanabe, Kiminobu Tanizawa, Kohei Ikezoe, Yoshio Taguchi, Hiroe Sato, Kazuo Chin, Sonoko Nagai, Shuh Narumiya, Athol U. Wells, Michiaki Mishima

**Affiliations:** 1 Department of Respiratory Medicine, Kyoto University Graduate School of Medicine, Kyoto, Japan; 2 Department of Rehabilitation Medicine, Kyoto University Hospital, Kyoto, Japan; 3 Department of Respiratory Care and Sleep Control Medicine, Kyoto University Graduate School of Medicine, Kyoto, Japan; 4 Department of Respiratory Medicine, Tenri Hospital, Tenri, Japan; 5 National Heart and Lung Institute, Imperial College London, London, United Kingdom; 6 Kyoto Central Clinic/Clinical Research Center, Kyoto, Japan; 7 Department of Pharmacology, Kyoto University Graduate School of Medicine, Kyoto, Japan; 8 Royal Brompton Hospital, London, United Kingdom; University of Pittsburgh, United States of America

## Abstract

**Background:**

Idiopathic pulmonary fibrosis (IPF) is a devastating lung disease of unknown etiology with few current treatment options. Recently, we determined an important role of prostaglandin F_2α_ (PGF_2α_) in pulmonary fibrosis by using a bleomycin-induced pulmonary fibrosis model and found an abundance of PGF_2α_ in bronchoalveolar lavage fluid of IPF patients. We investigated the role of PGF_2α_ in human IPF by assessing plasma concentrations of 15-keto-dihydro PGF_2α_, a stable metabolite of PGF_2α_.

**Methods:**

We measured plasma concentrations of 15-keto-dihydro PGF_2α_ in 91 IPF patients and compared these values with those of controls (n = 25). We further investigated the relationships of plasma 15-keto-dihydro PGF_2α_ concentrations with disease severity and mortality.

**Results:**

Plasma concentrations of 15-keto-dihydro PGF_2α_ were significantly higher in IPF patients than controls (*p*<0.001). Plasma concentrations of this metabolite were significantly correlated with forced expiratory volume in 1 second (*Rs* [correlation coefficient] = −0.34, *p* = 0.004), forced vital capacity (*Rs* = −0.33, *p* = 0.005), diffusing capacity for carbon monoxide (*Rs* = −0.36, *p* = 0.003), the composite physiologic index (*Rs* = 0.40, *p* = 0.001), 6-minute walk distance (*Rs* = −0.24, *p* = 0.04) and end-exercise oxygen saturation (*Rs* = −0.25, *p* = 0.04) when patients with emphysema were excluded. Multivariate analysis using stepwise Cox proportional hazards model showed that a higher composite physiologic index (relative risk = 1.049, *p* = 0.002) and plasma 15-keto-dihydro PGF_2α_ concentrations (relative risk = 1.005, *p* = 0.002) were independently associated with an increased risk of mortality.

**Conclusions:**

We demonstrated significant associations of plasma concentrations of PGF_2α_ metabolites with disease severity and prognosis, which support a potential pathogenic role for PGF_2α_ in human IPF.

## Introduction

Idiopathic pulmonary fibrosis (IPF) is a chronic, progressive, irreversible and usually lethal lung disease of unknown etiology that has limited therapeutic options [1]. Although IPF carries an overall poor prognosis [2], the clinical course of individual patients varies from slow progression to acute decompensation and death [3], [4]. A variety of pathways and mechanisms underlying pulmonary fibrosis have been identified [5], but have not been well evaluated in the clinical arena.

Prostaglandins (PGs) are oxygenated metabolites of arachidonic acid produced by sequential catalysis of cyclooxygenase (COX) and respective synthase, and they contribute to a variety of physiological responses and pathological processes [6]. PGE_2_ and PGI_2_ are considered to exert antifibrotic effects [7]. However, we recently reported that PGF_2α_ facilitates pulmonary fibrosis through PGF receptor (FP) in a murine bleomycin-induced pulmonary fibrosis model [8]. In addition, PGF_2α_ is abundant in bronchoalveolar lavage fluid of human subjects with IPF, and stimulates proliferation and collagen production of lung fibroblasts via FP [8]. This indicates that PGF_2α_ produced in the lung of IPF patients may contribute to disease progression.

Endogenous PGF_2α_ is swiftly degraded in various organs including the lung to 13,14-dihydro-15-keto PGF_2α_ (15-keto-dihydro PGF_2α_), a stable metabolite of PGF_2α_, which has a longer half-life in the circulation and has been used as a reliable indicator of *in vivo* PGF_2α_ biosynthesis [9]. In the current study, we measured plasma concentrations of this metabolite in a cohort of IPF patients to clarify their clinical relevance and prognostic value.

## Methods

### Ethics Statement

This study was approved by Kyoto University Graduate School and Faculty of Medicine Ethics Committee and the Ethics Committee of Tenri Hospital, and written informed consent was obtained from all patients.

### Study Subjects

The study population consisted of 91 IPF patients who visited Kyoto University Hospital from February 2008 through August 2011, and those who visited Tenri Hospital from April 2006 through September 2008. At the time of study entry, all of study patients had not been receiving any specific treatment for IPF.

IPF was diagnosed on the basis of the current official joint statement on IPF [10]. In 28 patients, usual interstitial pneumonia was confirmed by surgical lung biopsy. Quantification of disease severity by pulmonary function tests [11], and suitability of prognostic indicators [12] may be confounded by coexistent emphysema in patients with IPF. Therefore we also performed a subgroup analysis of patients without emphysema. Emphysema on high-resolution computed tomography was defined as the presence of well-demarcated areas of decreased attenuation in comparison with contiguous normal lung, and delimited by a very thin (<1 mm) or no wall, with upper zone predominance [13]. Age- and body mass index-matched subjects without lung disease (n = 25) were recruited as controls.

### Physiological Assessments

Pulmonary function tests were performed using CHESTAC system (Chest M.I. Inc., Tokyo, Japan). Diffusing capacity for carbon monoxide (DL_CO_) was measured using the single-breath technique. Percent-predicted values were used for analyses. The composite physiologic index (CPI) was calculated as previously described [11]. Arterial blood gas analysis, including arterial partial pressure of oxygen (PaO_2_) and arterial partial pressure of carbon dioxide (PaCO_2_), was performed while patients were breathing room air at rest in the supine position. The alveolar-arterial oxygen pressure difference (A-aDO_2_) was calculated according to a standard formula, using the respiratory exchange ratio of 0.8. Six-minute walk testing (6MWT) was performed as recommended by American Thoracic Society guidelines [14] and oxygen saturation was continuously monitored during 6MWT using a pulse oximeter (Pulsox-300i, Konica Minolta Inc., Osaka, Japan).

### Blood Sample Collection and Laboratory Assessments

Samples of peripheral venous blood were collected in the morning before breakfast. Plasma concentrations of 15-keto-dihydro PGF_2α_ were measured using an enzyme immunoassay kit (13,14-dihydro-15-keto Prostaglandin F_2α_ EIA kit; Cayman Chemical; Ann Arbor, MI, USA). Serum KL-6 levels were measured by a sandwich-type electrochemiluminescence immunoassay kit (Picolumi KL-6; Sanko Junyaku, Tokyo, Japan) and serum SP-D levels were measured by a sandwich-type enzyme immunoassay kit (SP-D kit Yamasa EIA II; Yamasa Shoyu, Chiba, Japan).

### Doppler Echocardiography

Twenty-eight IPF patients recruited in Kyoto University Hospital underwent Doppler echocardiography at study entry. Doppler echocardiography was performed using conventional clinical echocardiographic equipment (Xario XG, Toshiba Medical Systems Co., Ltd, Tochigi, Japan). Systolic pulmonary arterial pressure (sPAP) at rest was calculated by the sum of estimated right atrial pressure and the transtricuspid gradient as previously reported [15]. Pulmonary hypertension (PH) was defined as sPAP of greater than or equal to 40 mmHg at rest [15].

### Statistics

All statistical analyses were performed using JMP version 9 (SAS Institute, Cary, NC, USA). Continuous variables are expressed as mean±standard deviation. Comparisons of categorical data between two groups were performed by Fisher’s exact probability tests. Continuous variables were compared with the unpaired *t*-test if normally distributed, and the Mann-Whitney *U* test when the distribution was not normal. Correlations between pairs of variables were analyzed by Spearman’s rank correlation tests. For survival analysis, patients receiving lung transplantation during follow-up (n = 1) or subjects lost to follow-up (n = 10) were censored, and then the duration from entry to death, the transplant date, or the last visit was recorded. Mortality was first assessed for all risk factors using univariate Cox proportional hazard analysis, and then stepwise multivariate Cox proportional hazard analysis was performed to examine the prognostic predictive value of plasma 15-keto-dihydro PGF_2α_ concentrations while adjusting for other clinical predictors of cumulative mortality. Results of the regression analysis were presented in terms of relative risks (RRs) with corresponding 95% confidence intervals. Survival curves were obtained using the Kaplan-Meier method and the difference in survival rates between subgroups was calculated using a log-rank test. A *p* value less than 0.05 was considered to indicate statistical significance.

## Results

### Plasma 15-keto-dihydro PGF_2α_ Concentrations Correlate with Disease Severity Indices

**Figure 1:**
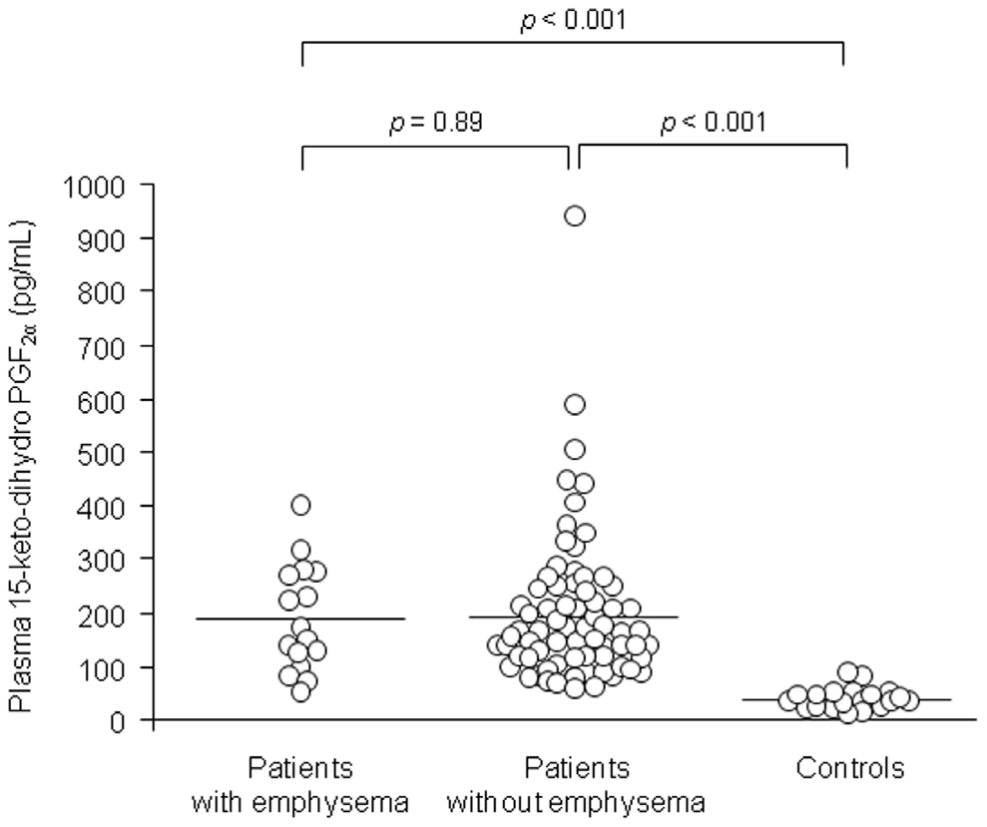
Comparison of plasma 15-keto-dihydro prostaglandin F_2α_ concentrations in patients and controls. Horizontal bars indicate mean values. There were significant differences in plasma 15-keto-dihydro prostaglandin F_2α_ concentrations between IPF patients with emphysema (n = 16) and controls (n = 25), and IPF patients without emphysema (n = 75) and controls. However, there was no difference in plasma 15-keto-dihydro prostaglandin F_2α_ concentrations between IPF patients with emphysema and those without emphysema.

The clinical characteristics of patients and controls are summarised in Table 1 (see Table S1 for the patient characteristics in each hospital). The proportions of females (*p* = 0.03) and never smokers (*p*<0.001) were significantly lower in IPF patients than those in control subjects. Among all 91 patients, 16 had concurrent emphysema. Plasma concentrations of 15-keto-dihydro PGF_2α_ were significantly higher in IPF patients than those in control subjects, regardless of the presence of concurrent emphysema (Figure 1, *p*<0.001).

**Table 1:** Characteristics of patients and controls.

	All patients (n = 91)	Emphysemaexcluded (n = 75)	Controls (n = 25)	*p* value*
Sex, male/female	69/22	57/18	13/12	0.03
Age, years	66.5±7.9	66.0±7.8	64.6±6.4	0.27
BMI, kg/m^2^	23.4±3.0	23.4±3.0	23.2±2.2	0.80
Nonsmokers	15	14	16	<0.001
Disease duration**, months	16.0±25.5	16.3±25.4	NA	
FEV_1_, % predicted	98.6±21.0	98.5±21.6	NA	
FVC, % predicted	89.2±22.1	87.6±20.9	NA	
DL_CO_, % predicted	52.6±17.7	52.1±16.5	NA	
Composite physiologic index	43.8±14.0	43.7±13.9	NA	
PaCO_2_, kPa	5.7±0.9	5.7±0.8	NA	
PaO_2_, kPa	10.9±1.7	11.3±1.6	NA	
A-aDO_2_, kPa	1.4±1.9	1.2±1.7	NA	
Six-minute walk distance, m	449.1±92.0	454.9±87.2	NA	
End-exercise oxygen saturation, %	87.9±6.9	88.7±6.4	NA	
Serum KL-6, U/mL	1135±842	1112±829	NA	
Serum SP-D, ng/mL	253±211	263±199	NA	
Plasma15-keto-dihydro PGF_2α_, pg/mL	193±133	194±140	37±19	<0.001

Data are presented as mean±standard deviation.

* Comparison between all patients and controls.

** Time from diagnosis to blood sample collection.

BMI, body mass index; FEV_1_, forced expiratory volume in 1 second; FVC, forced vital capacity; DL_CO_, diffusing capacity for carbon monoxide; PaCO_2_, arterial partial pressure of carbon dioxide; PaO_2_, arterial partial pressure of oxygen; A-aDO_2_, alveolar-arterial oxygen pressure difference; SP-D, surfactant protein-D; PGF_2α_, prostaglandin F_2α_; NA, not available.

**Figure 2:**
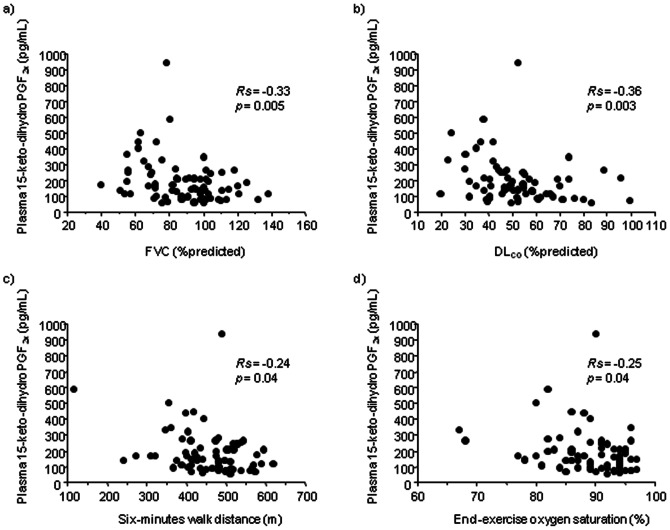
Correlations of plasma 15-keto-dihydro prostaglandin F_2α_ concentrations with indices of disease severity. Scatter diagrams show the correlations of plasma 15-keto-dihydro prostaglandin F_2α_ concentrations with FVC (a), DL_CO_ (b), six-minute walk distance (c) and end-exercise oxygen saturation (d) in IPF patients without emphysema. The *Rs* value indicates the correlation coefficient.

To assess the clinical relevance of plasma 15-keto-dihydro PGF_2α_ concentrations, we investigated their relationships with disease duration, pulmonary function, arterial blood gas data and 6MWT data (Table 2). Spearman’s rank correlation analysis showed that plasma concentrations of 15-keto-dihydro PGF_2α_ were significantly correlated with forced expiratory volume in 1 second (FEV_1_) (*Rs* [correlation coefficient] = −0.22, *p* = 0.03) but not with disease duration or other indices of disease severity. However, among patients without emphysema (n = 75), plasma concentrations of 15-keto-dihydro PGF_2α_ were significantly correlated with FEV_1_ (*Rs* = −0.34, *p* = 0.004), forced vital capacity (FVC) (*Rs* = −0.33, *p* = 0.005), DL_CO_ (*Rs* = −0.36, *p* = 0.003), CPI (*Rs* = −0.40, *p* = 0.001), A-aDO_2_ (*Rs* = 0.39, *p* = 0.002), 6-minute walk distance (*Rs* = −0.24, *p* = 0.04) and end-exercise oxygen saturation (*Rs* = −0.25, *p* = 0.04) (Figure 2).

**Table 2:** Relationships between plasma 15-keto-dihydro prostaglandin F_2α_ concentrations and clinical parameters in patients with idiopathic pulmonary fibrosis.

	All patients (n = 91)		Emphysema excluded (n = 75)	
	Correlation coefficient	*p* value	Correlation coefficient	*p* value
Disease duration*, months	−0.04	0.20	0.03	0.80
**Pulmonary function**				
FEV_1_, % predicted	−0.22	0.03	−0.34	0.004
FVC, % predicted	−0.11	0.28	−0.33	0.005
DL_CO_, % predicted	−0.16	0.13	−0.36	0.003
Composite physiologic index	0.19	0.08	0.40	0.001
**Arterial blood gas data**				
PaCO_2_, kPa	−0.09	0.40	−0.13	0.30
PaO_2_, kPa	−0.14	0.22	−0.24	0.05
A-aDO_2_, kPa	0.21	0.06	0.39	0.002
**Six-minute walk test**				
Six-minute walk distance, m	−0.21	0.06	−0.24	0.04
End-exercise oxygen saturation, %	−0.14	0.20	−0.25	0.04

* Time from diagnosis to blood sample collection.

FEV_1_, forced expiratory volume in 1 second; FVC, forced vital capacity; DL_CO_, diffusing capacity for carbon monoxide; PaCO_2_, arterial partial pressure of carbon dioxide; PaO_2_, arterial partial pressure of oxygen; A-aDO_2_, alveolar-arterial oxygen pressure difference.

### Plasma 15-keto-dihydro PGF_2α_ Concentrations Predict Mortality

During a mean follow-up period of 29.8 (range: 0.5–74.9) months, 22 of 91 patients died. The major cause of death was respiratory failure due to the progression of IPF (n = 15), 4 patients died of lung cancer and 3 patients died suddenly at home from an unknown cause. Although none of the patients received IPF-specific treatment at entry, some underwent treatment with pirfenidone (n = 13), prednisone (n = 25), cyclophosphamide (n = 3), azathioprine (n = 4) and cyclosporine (n = 6) during a follow-up period. Combination therapy with two or more medications was used in 14 patients.

We assessed the relationships between clinical measurements and mortality using Cox proportional hazard model (Table 3). Among overall patients (n = 91) as well as patients without emphysema (n = 75), low FEV_1_, low FVC, low DL_CO_, high CPI, short 6-minute walk distance and low end-exercise oxygen saturation were significantly associated with an increased risk of mortality. Plasma concentrations of 15-keto-dihydro PGF_2α_ were significantly associated with mortality (RR = 1.005, *p*<0.001), but sex, age and serum levels of KL-6 and SP-D were not. Multivariate analysis was performed using stepwise Cox proportional hazard model in which all significant predictors of mortality in univariate analyses were included as covariates. We found that high CPI (RR = 1.049, *p* = 0.002 in all patients; RR = 1.043, *p* = 0.016 in patients without emphysema) and increased plasma concentrations of 15-keto-dihydro PGF_2α_ (RR = 1.005, *p* = 0.002 in all patients; RR = 1.005, *p* = 0.003 in patients without emphysema) were the independent predictors of mortality. The independent association of plasma 15-keto-dihydro PGF_2α_ concentrations with mortality was reproducibly found in each cohort (see Table S2).

**Table 3:** Cox proportional hazard model results for evaluating the risk of mortality.

	All patients (n = 91)			Emphysema excluded (n = 75)		
	Relative risk	95% CI	*p* value	Relative risk	95% CI	*p* value
**Univariate analysis**						
Female sex	0.386	0.090–1.667	0.20	0.372	0.086–1.613	0.19
Age, years	1.028	0.964–1.095	0.40	1.036	0.965–1.112	0.32
Smoking, pack-years	0.997	0.984–1.010	0.61	1.000	0.988–1.012	0.94
FEV_1_, % predicted	0.972	0.949–0.996	0.02	0.974	0.950–0.998	0.03
FVC, % predicted	0.963	0.941–0.986	0.002	0.961	0.938–0.985	0.002
DL_CO_, % predicted	0.952	0.922–0.983	0.003	0.956	0.927–0.986	0.005
Composite physiologic index	1.060	1.022–1.099	0.002	1.059	1.021–1.099	0.002
Six-minute walk distance, m	0.994	0.990–0.999	0.02	0.991	0.986–0.997	0.002
End-exercise oxygen saturation, %	0.908	0.852–0.969	0.004	0.878	0.819–0.943	<0.001
Serum KL-6, U/mL	1.000	1.000–1.001	0.52	1.000	1.000–1.001	0.63
Serum SP-D, ng/mL	1.001	0.999–1.003	0.46	1.002	0.999–1.004	0.22
Plasma 15-keto-dihydro PGF_2α_, pg/mL	1.005	1.002–1.007	<0.001	1.005	1.002–1.007	<0.001
**Multivariate analysis**						
Composite physiologic index	1.049	1.016–1.088	0.002	1.043	1.008–1.084	0.016
Plasma 15-keto-dihydro PGF_2α_, pg/mL	1.005	1.002–1.007	0.002	1.005	1.002–1.008	0.003

CI, confidence interval; FEV_1_, forced expiratory volume in 1 second; FVC, forced vital capacity; DL_CO_, diffusing capacity for carbon monoxide; SP-D, surfactant protein-D; PGF_2α_, prostaglandin F_2α_.

Figure S1 shows the Kaplan-Meier survival curves when patients were divided into two groups, with plasma 15-keto-dihydro PGF_2α_ concentrations above or below the median value (156 pg/mL), respectively. Patients with higher plasma 15-keto-dihydro PGF_2α_ concentrations (≥156 pg/mL) had a significantly worse prognosis than patients with lower concentrations (*p* = 0.04).

### Plasma 15-keto-dihydro PGF_2α_ Concentrations are Associated with the Presence of Pulmonary Hypertension Assessed by Doppler Echocardiography

Finally, since PH is a significant comorbidity which can affect prognosis in patients with IPF [16], [17], we compared plasma 15-keto-dihydro PGF_2α_ concentrations according to the presence or absence of PH. Among patients with successful sPAP measurements, 8 of 28 had sPAP≥40 mmHg, which fulfilled the definition of PH. Plasma concentrations of 15-keto-dihydro PGF_2α_ were significantly higher in patients with PH than in those without PH (317±291 pg/mL vs 172±75 pg/mL, *p* = 0.04). However, there were no significant differences in serum levels of KL-6 and SP-D between patients with and without PH (1503±444 U/mL vs 1252±735 U/mL, *p* = 0.38; and 367±181 ng/mL vs 347±314 ng/mL, *p* = 0.87, respectively).

## Discussion

We demonstrated raised concentrations of 15-keto-dihydro PGF_2α_, a stable metabolite of PGF_2α_, in the plasma of patients with IPF. Plasma concentrations of this metabolite were significantly correlated with indices of disease severity including FEV_1_, FVC, DL_CO_, CPI, 6-minute walk distance and end-exercise oxygen saturation, when patients with emphysema were excluded. Furthermore, in our cohort of patients, higher plasma 15-keto-dihydro PGF_2α_ concentrations were significantly associated with an increased risk of mortality after adjusting for disease severity indices represented by CPI. Among patients with successful echocardiographic measurements, plasma 15-keto-dihydro PGF_2α_ concentrations were significantly higher in patients with PH than in those without PH.

Considering that IPF is a devastating lung disease with poor prognosis but highly variable clinical course, predicting which paths individual patients will take remains a central challenge for clinicians [18]. A primary finding of the present study was that plasma concentrations of PGF_2α_ metabolite were significantly correlated with the severity of IPF as well as mortality. However, the relationships of plasma 15-keto-dihydro PGF_2α_ concentrations with pulmonary function, arterial blood gas data and 6MWT data differed according to the presence of concomitant emphysema, suggesting that emphysema is a significant confounding comorbidity in IPF. FVC, DL_CO_, CPI and desaturation during 6MWT predicted mortality in our cohort of patients with IPF, which is consistent with previous studies [11], [17], [19], [20]. However, even after adjusting for these physiologic predictors, plasma 15-keto-dihydro PGF_2α_ concentrations were significantly associated with mortality. We have recently shown by both *in vivo* and *in vitro* approaches that PGF_2α_-FP signaling mediates pulmonary fibrosis independently of transforming growth factor-β signaling [8], which is a well-known crucial pathway for fibrogenesis [21]. Raised concentrations of plasma PGF_2α_ metabolite may reflect an upregulation of PGF_2α_-FP signaling pathway and subsequent fibrotic processes, which cannot be evaluated by a single measurement of pulmonary function or 6MWT. Indeed, the relationships between plasma concentration of PGF_2α_ metabolite and physiological parameters of IPF tended to be significant but relatively weak.

Another possible explanation why plasma PGF_2α_ metabolite concentrations predicted mortality was the impact of comorbid PH. In subgroup analysis, plasma PGF_2α_ metabolite concentrations were significantly higher in patients with PH, whereas serum levels of KL-6 and SP-D did not differ between the subgroups. Since PH commonly complicates the course of IPF and potentially affects prognosis [16], [17], the significant relationship of plasma PGF_2α_ metabolite concentrations with mortality may also reflect a higher frequency of concomitant PH in patients with higher plasma concentrations of PGF_2α_ metabolite.

Since PGF_2α_ is a locally bioactive hormone that is uncovered virtually in all tissues including lung [9], the source of PGF_2α_ or its metabolite in the plasma of pulmonary fibrosis patients is speculative. For example, macrophages activated by proinflammatory stimuli produce PGF_2α_ in large amounts [22]. PGF_2α_ is also produced by type II alveolar epithelial cells [23], and widely upregulated COX-2 expression was observed in metaplastic epithelium in pulmonary fibrous disorders [24]. In addition, PGF synthase exists in contractile interstitial cells of bovine lungs, which are considered to be a precursor of myofibroblasts, main contributing cell types to fibrogenesis [25]. Thus, increased epithelial permeability in the lung of IPF patients [26] may allow PGF_2α_ produced in the lungs to leak into the circulation, where it is instantly bioconverted through metabolism [9].

Another possible mechanism might be functional alteration in pulmonary arterial endothelium and/or smooth muscle cells, both of which play significant roles in the pathogenesis of pulmonary arterial hypertension [27]. Recent studies suggested that the pathogenesis of PH in IPF is a complicated interaction of epithelial cells, fibroblasts and vascular cells mediated by multiple factors including several growth factors [28], which can stimulate the expression of COX-2, thereby leading to the production of PGs [29]. The COX-2 pathway might be upregulated in pulmonary arterial endothelium and/or smooth muscle cells through hypoxic stimulus and/or the development of PH. This pathogenesis is worthy to be studied further because, in contrast, previously identified biomarkers of IPF, KL-6 and SP-D, were found to be mainly produced by alveolar epithelial cells [30], [31].

Cytosolic phospholipase A_2_ (cPLA_2_), which cleaves phospholipids and yields arachidonic acids including PGs as well as lysophospholipids, might play a pivotal role in the pathogenesis of pulmonary fibrosis [32]. Thus, PGF_2α_ and lysophosphatidic acids, both derived from the breakdown of phospholipids by cPLA_2_ and possible stimulators of fibrosis [8], [33], might act complementarily to affect the progression of disease.

The present study has some limitations. Firstly, this is a relatively small cohort study with IPF patients alone. Since PGF_2α_ is produced by various cells and is implicated in the regulation of intricate pathophysiological processes [9], further studies on the utility of plasma 15-keto-dihydro PGF_2α_ in patients with systemic disease-associated interstitial lung diseases are warranted. At the time of blood sampling, all study participants were not receiving any specific treatment for IPF. Hence the possible effects of IPF-specific medication on the value and usefulness of plasma 15-keto-dihydro PGF_2α_ should also be assessed for future clinical use. Secondly, since the presence and degree of PH were not evaluated by right heart catheterisation and echocardiographic data were not available in all patients, our current findings should be interpreted with caution and further studies are necessary.

In summary, we demonstrated significant associations of plasma concentrations of PGF_2α_ metabolites with disease severity and prognosis in IPF patients. Although further larger studies are needed to confirm the clinical utility of this metabolite, the current findings support a potential pathogenic role for PGF_2α_ in human IPF. Our current results also provide a more integrated understanding of this devastating and complex disease and may help in the development of new antifibrotic drugs.

## Supporting Information

Figure S1 **Kaplan-Meier survival analysis grouped by baseline plasma 15-keto-dihydro prostaglandin F_2α_ concentrations.** The *black line* represents the group of IPF patients with baseline plasma 15-keto-dihydro prostaglandin F_2α_ concentrations greater than or equal to 156 pg/mL. The *gray line* represents the group of IPF patients with baseline plasma 15-keto-dihydro prostaglandin F_2α_ concentrations less than 156 pg/mL.
(TIF)

Table S1 Patient characteristics of two cohorts.
(DOC)

Table S2 Cox proportional hazard model results for evaluating the risk of mortality.
(DOC)
