# Supplementary figures and images for: Clinical Relevance of Plasma Prostaglandin F2α Metabolite Concentrations in Patients with Idiopathic Pulmonary Fibrosis
Source: PLoS One. 2013 Jun 11;8(6):e66017. doi: 10.1371/journal.pone.0066017 (PMC3679025; doi:10.1371/journal.pone.0066017)

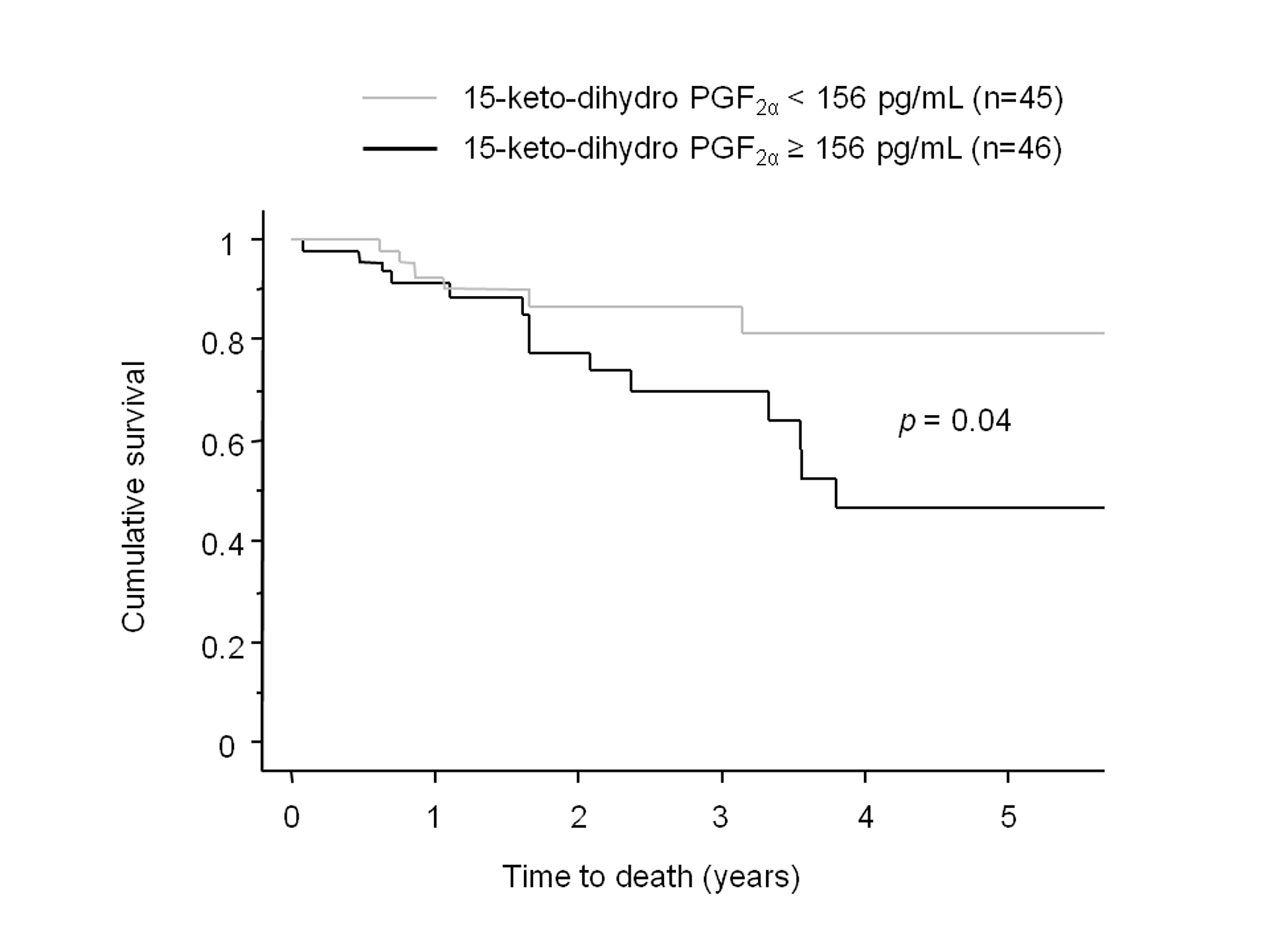

Supplement: Figure S1 — Kaplan-Meier survival analysis grouped by baseline plasma 15-keto-dihydro prostaglandin F2α concentrations. The black line represents the group of IPF patients with baseline plasma 15-keto-dihydro prostaglandin F2α concentrations greater than or equal to 156 pg/mL. The gray line represents the group of IPF patients with baseline plasma 15-keto-dihydro prostaglandin F2α concentrations less than 156 pg/mL. (TIF) [file pone.0066017.s001.tif]
