# Supplementary material for: Clinical Relevance of Plasma Prostaglandin F2α Metabolite Concentrations in Patients with Idiopathic Pulmonary Fibrosis
Source: PLoS One. 2013 Jun 11;8(6):e66017. doi: 10.1371/journal.pone.0066017 (PMC3679025; doi:10.1371/journal.pone.0066017)
Supplement: Table S1 — Patient characteristics of two cohorts. (DOC) [file pone.0066017.s002.doc]

**Table S1.** Patient characteristics of two cohorts.

|  | Kyoto University Hospital (n=52) | Tenri Hospital (n=39) | *p* value |
| --- | --- | --- | --- |
| Sex, male/female | 39/13 | 30/9 | 0.99 |
| Age, years | 64.9±8.4 | 68.5±6.8 | 0.03 |
| BMI, kg/m^2^ | 23.5±3.0 | 23.3±3.2 | 0.78 |
| Nonsmokers, n | 8 | 7 | 0.78 |
| Disease duration^*^, months | 15.4±20.2 | 16.8±31.4 | 0.07 |
| FEV_1_, % predicted | 97.4±22.3 | 100.1±19.4 | 0.55 |
| FVC, % predicted | 88.4±22.3 | 90.3±22.1 | 0.68 |
| DL_CO_, % predicted | 48.1±12.6 | 58.9±21.7 | 0.005 |
| Composite physiologic index | 45.7±12.1 | 41.1±2.8 | 0.15 |
| PaCO_2_, kPa | 5.5±0.4 | 5.9±1.2 | 0.04 |
| PaO_2_, kPa | 11.2±1.4 | 10.6±2.1 | 0.14 |
| A-aDO_2_, kPa | 1.9±1.5 | 0.8±2.1 | 0.01 |
| Six-minute walk distance, m | 451±76 | 446±115 | 0.82 |
| End-exercise oxygen saturation, % | 89.5±5.2 | 85.1±8.5 | 0.006 |
| Serum KL-6, U/mL | 1228±917 | 1011±724 | 0.22 |
| Serum SP-D, ng/mL | 315±242 | 154±79 | <0.001 |
| Plasma 15-keto-dihydro PGF_2α_, pg/mL | 202±148 | 182±111 | 0.48 |

Data are presented as mean±standard deviation.

* Time from diagnosis to blood sample collection.

BMI, body mass index; FEV_1_, forced expiratory volume in 1 second; FVC, forced vital capacity; DL_CO_, diffusing capacity for carbon monoxide; PaCO_2_, arterial partial pressure of carbon dioxide; PaO_2_, arterial partial pressure of oxygen; A-aDO_2_, alveolar-arterial oxygen pressure difference; SP-D, surfactant protein-D; PGF_2α_, prostaglandin F_2α_.
