# Supplementary material for: Clinical Relevance of Plasma Prostaglandin F2α Metabolite Concentrations in Patients with Idiopathic Pulmonary Fibrosis
Source: PLoS One. 2013 Jun 11;8(6):e66017. doi: 10.1371/journal.pone.0066017 (PMC3679025; doi:10.1371/journal.pone.0066017)
Supplement: Table S2 — Cox proportional hazard model results for evaluating the risk of mortality. (DOC) [file pone.0066017.s003.doc]

**Table S2.** Cox proportional hazard model results for evaluating the risk of mortality.

|  | Kyoto University Hospital (n=52) | | | Tenri Hospital (n=39) | | |
| --- | --- | --- | --- | --- | --- | --- |
|  | Relative risk | 95% CI | *p* value | Relative risk | 95% CI | *p* value |
| **Univariate analysis** |  |  |  |  |  |  |
| Female sex | 0.410 | 0.052-3.247 | 0.40 | NA | NA | NA |
| Age, years | 1.047 | 0.976-1.123 | 0.20 | 1.004 | 0.893-1.130 | 0.95 |
| FEV_1_, % predicted | 0.975 | 0.941-1.011 | 0.17 | 0.971 | 0.935-1.009 | 0.13 |
| FVC, % predicted | 0.961 | 0.928-0.996 | 0.03 | 0.961 | 0.925-0.997 | 0.04 |
| DL_CO_, % predicted | 0.938 | 0.881-0.998 | 0.04 | 0.959 | 0.920-0.999 | 0.04 |
| Composite physiologic index | 1.071 | 1.006-1.141 | 0.03 | 1.054 | 1.002-1.108 | 0.04 |
| Six-minute walk distance, m | 0.996 | 0.990-1.002 | 0.16 | 0.995 | 0.987-1.003 | 0.22 |
| End-exercise oxygen saturation, % | 0.881 | 0.784-0.990 | 0.03 | 0.884 | 0.790-0.990 | 0.03 |
| Serum KL-6, U/mL | 1.000 | 0.999-1.001 | 0.99 | 1.000 | 0.999-1.001 | 0.68 |
| Serum SP-D, ng/mL | 1.000 | 0.998-1.003 | 0.91 | 0.992 | 0.977-1.007 | 0.30 |
| Plasma 15-keto-dihydro PGF_2α_, pg/mL | 1.005 | 1.002-1.009 | 0.002 | 1.007 | 1.001-1.013 | 0.02 |
| **Multivariate analysis** |  |  |  |  |  |  |
| Composite physiologic index | 1.022 | 0.957-1.090 | 0.52 | 1.097 | 1.026-1.174 | 0.007 |
| Plasma 15-keto-dihydro PGF_2α_, pg/mL | 1.005 | 1.001-1.010 | 0.02 | 1.015 | 1.004-1.027 | 0.008 |

CI, confidence interval; FEV_1_, forced expiratory volume in 1 second; FVC, forced vital capacity; DL_CO_, diffusing capacity for carbon monoxide; SP-D, surfactant protein-D; PGF_2α_, prostaglandin F_2α_; NA, not available.
